# Supplementary material for: Analysis of PPARγ Signaling Activity in Psoriasis
Source: Int J Mol Sci. 2021 Aug 10;22(16):8603. doi: 10.3390/ijms22168603 (PMC8395241; doi:10.3390/ijms22168603)
Supplement: Supplementary file 1 [file ijms-22-08603-s001.zip › Supplemental materials_Analysis of PPARg signaling activity in psoriasis/Pathway models/Models images and html files/Model of PPARG signaling in psoriasis/13301.html]

TRADD


# Protein TRADD

|  |  |
| --- | --- |
| URN | urn:agi-llid:8717 |
| Total Entities | 0 |
| Connectivity | 573 |
| Name | TRADD |
| Description | TNFRSF1A-associated via death domain |
| Notes | The protein encoded by this gene is a death domain containing adaptor molecule that interacts with TNFRSF1A/TNFR1 and mediates programmed cell death signaling and NF-kappaB activation. This protein binds adaptor protein TRAF2, reduces the recruitment of inhibitor-of-apoptosis proteins (IAPs) by TRAF2, and thus suppresses TRAF2 mediated apoptosis. This protein can also interact with receptor TNFRSF6/FAS and adaptor protein FADD/MORT1, and is involved in the Fas-induced cell death pathway. [provided by RefSeq, Jul 2008] |
| Primary Cell Localization | Cytoplasm |

---

|  |  |
| --- | --- |
| Pathway | Osteoarthritis |
|  | Skin Aging |
|  | MTHFR Activity Regulation |
|  | mTOR Signaling Hyperfunction |
|  | TLR4 Signaling in Leukocytes |
|  | Peripheral T-Cell Tolerance |
|  | T-Cell Cytotoxic Mediated Cell Death |
|  | Necrosis |
|  | Leukotriene Effect on Vascular Endothelial Cell Response |
|  | Eosinophil Survival by Cytokine Signaling |
|  | Macrophage M1 Lineage |
|  | Neutrophil Recruitment and Priming |
|  | Negative Acute Phase Proteins Synthesis |
|  | Positive Acute Phase Proteins Synthesis |
|  | Vascular Endothelial Cell Activation by Cytokines |
|  | Adipokines Production by Adipocyte |
|  | Lipolysis Regulation in Adipocyte |
|  | GFs/TNF -> Ion Channels |
|  | Circadian Clock Genes in Suprachiasmatic Nuclei Neurons |
|  | Apoptosis |
|  | Dilated Cardiomyopathy |
|  | Cardiomyocyte Hypertrophy |
|  | Hypertrophic Cardiomyopathy |
|  | Myocardial Infarction |
|  | Apoptosis of Myocyte |
|  | Inflammatory Reaction in Acne Vulgaris |
|  | Th0-Cell Aberrant Activation in Systemic Lupus Erythematosus |
|  | Vitiligo |
|  | Apoptosis of Melanocyte in Vitiligo |
|  | Toll-like Receptors in beta-Cell |
|  | Podocyte Dysfunction in Diabetic Nephropathy |
|  | Adipose Tissue Inflammation Provokes Insulin Resistance |
|  | Oxidation of Fatty Acid in non-Alcoholic Fatty Liver Disease |
|  | beta-Cell Destruction through Cytokines in Diabetes Mellitus |
|  | beta-Cell Destruction in Diabetes Mellitus Type 1 |
|  | Adipokines Production by Adipocyte Impaired in Obesity |
|  | Crohn's Disease |
|  | Macrophage Dysfunction |
|  | T-Cell Cytotoxic Response in Ulcerative Colitis |
|  | TNFR/NF-kB Classical Pathway in Multiple Myeloma Plasma Cells |
|  | Multiple Myeloma |
|  | Eosinophil Survival in Asthma |
|  | Goblet-Cell Related Mucus Secretion in Asthma |
|  | Club-Cell Role in Asthma |
|  | Airway Epithelial Cell Dysfunction |
|  | Neutrophil Recruitment in Sinovium in Gout |
|  | TNF and IL1B Induce Metalloproteinase Synthesis in Osteoarthritis |
|  | Apoptosis of Chondrocyte |
|  | Dendritic Cell Dysfunction in Psoriatic Arthritis |
|  | Synovial Fibroblast Activation in Psoriatic Arthritis |
|  | Synovial Fibroblast Activation by Citokines in Rheumatoid Arthritis |
|  | Apoptosis Block in Synovial Fibroblast |
|  | Skin Fibrosis |
|  | Dentin and Enamel Dysruprion in Bloch-Sulzberger Syndrome |
|  | Apoptosis Block in Infected Cells |
|  | Diabetes Induced Periodontitis |
|  | IL1 Receptor Antagonist (IL1RN) Deficiency (DIRA) |
|  | TNF Receptor Associated Periodic Syndrome |
|  | Osteoclast Activation in Postmenopause |
|  | Proteins Involved in Alzheimer's Disease |
|  | Apoptosis and Immediate Early Gene Activation in Epileptiform Disorders |
|  | Glioblastoma, Mesenchymal Subtype |
|  | Growth Factor Signaling in Neuroblastoma |
|  | Neuroblastoma |
|  | Crystallin alpha B (CRYAB) Suppresses Multiple Sclerosis |
|  | Breast Cancer |
|  | Subepithelial Fibroblasts in Middle Ear Cholesteatoma |
|  | HPV E6 and E7 Induced Disruption of TNFR/NF-kB Signaling in Keratinocytes |
|  | OPTN Effects on NF-kB Signaling in Glaucoma |
|  | Neurotrophic Factor Deprivation in Retinal Ganglion Cell Death |
|  | Alveolar Macrophages Dysfunction in COPD |
|  | Mucin Hyperproduction in Goblet and Mucous Cells |
|  | Podocyte Dysfunction in Glomerulonephritis |
|  | Interstitial Fibroblasts in Pyelonephritis |
|  | Apoptosis of Renal Tubule Epithelial Cell in Pyelonephritis |
|  | NFKB Canonical Signaling Activation in Cancer |
|  | STAT3 and NFkB Activate Inflammation-Induced Tumorigenesis |
|  | NFKB Signaling Activation by Blocking of Tumor Suppressors |
|  | Proteins with Altered Expression in Cancer-Associated Resisting to Cell Death |
|  | Apoptosis Block by Death Receptor Signaling in Cancer |
|  | Apoptosis Evasion in Cancer: Overview |
|  | Cancer Cells Inhibit Adipocyte Differentiation |
|  | Skeletal Muscle Wasting in Cancer Cachexia |
|  | IDO1 in Cancer Immune Escape |
|  | Effector T-cell Inactivation in Cancer Immune Escape |
|  | NGF -> SMAD3/NF-kB Expression Targets |
|  | NGF -> AP-1/TP53/MYC Expression Targets |
|  | NGF -> FOXO/MYCN/ELK-SRF Expression Targets |
|  | BDNF Expression Targets |
|  | NTF3 Expression Targets |
|  | NTF4 Expression Targets |
|  | NGF -> CREB/CEBPB/MEF2A Expression Targets |
|  | TNF-alpha/TNFRSF1A Signaling |
|  | NF-kB Canonical Signaling |
|  | FASLG Expression Targets |
|  | TNF -> NF-kB Expression Targets |
|  | TNFSF10 Expression Targets |
|  | LTA Expression Targets |
|  | TNF -> AP-1 Expression Targets |
|  | TNF -> ELK-SRF Expression Targets |
|  | TNF -> CREB Expression Targets |
|  | TNF -> TP53/ATF Expression Targets |
|  | TNFRSF6 -> FOXO3A Signaling |
|  | TNFR -> CREB/ELK-SRF Signaling |
|  | TNFR -> AP-1/ATF/TP53 Signaling |
|  | NGFR -> AP-1/CEBPB/CREB/ELK/SRF/TP53 Signaling |
|  | TNFRSF5/6 -> RB1/E2F Signaling |
|  | NGFR -> NF-kB Signaling |
|  | TNFR -> NF-kB Signaling |
|  | NGFR -> MEF Signaling |
|  | TNFRSF6 -> DDIT3 Signaling |
|  | TNFRSF6 -> HSF1 Signaling |
|  | TNFRSF1A -> AP-1/ATF/TP53 Signaling |
|  | TNFRSF1A -> CREB/ELK-SRF Signaling |
|  | Model of PPARG signaling in psoriasis |
|  | 1\_Inflammation in keratinocytes in the pathology of acne |
|  | 1\_TNF and IL1B provoke ECM degradation in osteoarthritis |
|  | 1\_Osteoclast-mediated bone resorption in postmenopausal women |
|  | 2\_Chondrocyte death in osteoarthritis |
|  | 3\_The role of interstitial fibroblasts in pyelonephritis |
|  | 2\_Podocyte dysfunction in glomerulonephritis |
|  | 3\_Role of neurotrophic factor deprivation in retinal ganglion cell death |
|  | 2\_Pathogens stimulate mucins expression in the middle ear |
|  | 2\_Cardiomyocyte hypertrophy |
|  | 3\_Role of alveolar macrophages in pulmonary tissue destruction in COPD |
|  | 3\_2\_Regulation of airway surface liquid (ASL) thickness |
|  | 3\_1\_Mucus accumulation in asthma |
|  | 2\_2\_Eosinophilia and neutrophilia in asthma: eosinophil apoptosis |
|  | 4\_Mucin hyperproduction in goblet and mucous cells in COPD |
|  | 2\_Lipotoxicity in NAFLD |
|  | 6\_p12I Viral protein promotes T-cell activation in HTLV-1 infection |
|  | 5\_Human papilloma virus E6 and E7 proteins promote epithelial cell proliferation |
|  | 2\_HIV - induced depletion of CD4+ T-cells |
|  | 4\_Inflammatory mediators inhibit GATA signaling in erythropoiesis |
|  | 4\_2\_Insulin resistance: inflammation-related insulin resistance |
|  | 1\_Antigen-presenting cells promote islet dysfunction and immune system activation in T1D |
|  | 3\_Islet beta-cell destruction in T1D |
|  | 2\_Neuron and Schwann cell death |

---

|  |  |
| --- | --- |
| MedScan ID | 8717 |

---

|  |  |
| --- | --- |
| LocusLink ID | 8717 |
|  | 71609 |
|  | 246756 |
|  | 22028 |
|  | 101978 |
|  | 110684 |

---

|  |  |
| --- | --- |
| Alias | TNFR1-associated DEATH domain protein |
|  | TNF receptor-associated death domain protein |
|  | MGC11078 |
|  | tumor necrosis factor receptor-1-associated protein |
|  | TNF receptor 1 associated signal transducer |
|  | 9130005N23Rik |
|  | Tumor necrosis factor receptor type 1 associated DEATH domain protein |
|  | TNF receptor associated death domain protein |
|  | AA930854 |
|  | Hs.89862 |
|  | tumor necrosis factor receptor associated death domain protein |
|  | LOC123915 |
|  | TRADD |
|  | 9130005N23 |
|  | TNFR-associated DD protein |
|  | TNFRSF1A-associated via death domain |
|  | TNFR-associated DEATH domain protein |
|  | tumor necrosis factor receptor type 1-associated DEATH domain protein |
|  | tumour necrosis factor receptor associated death domain protein |
|  | tumour necrosis factor receptor-1-associated protein |
|  | tumour necrosis factor receptor type 1 associated DEATH domain protein |
|  | RIKEN cDNA 9130005N23 gene |

---

|  |  |
| --- | --- |
| GO ID | 0070513 |
|  | 0019900 |
|  | 0044877 |
|  | 0005068 |
|  | 0005164 |
|  | 0007249 |
|  | 0006919 |
|  | 0006915 |
|  | 0071356 |
|  | 0071550 |
|  | 0097191 |
|  | 0008625 |
|  | 1902042 |
|  | 0043123 |
|  | 0051092 |
|  | 1901224 |
|  | 0043065 |
|  | 0030335 |
|  | 0051798 |
|  | 0050729 |
|  | 0051291 |
|  | 1902041 |
|  | 0010803 |
|  | 0007165 |
|  | 0007169 |
|  | 0033209 |
|  | 0005737 |
|  | 0005856 |
|  | 0005829 |
|  | 0031264 |
|  | 0045121 |
|  | 0005634 |
|  | 0005886 |
|  | 0043235 |
|  | 0060090 |
|  | 0042981 |
|  | 0032991 |
|  | 0032403 |
|  | 0004871 |
|  | 0097296 |
|  | 0010939 |
|  | 0043234 |
|  | 2001239 |
|  | 0097190 |
|  | 0008633 |
|  | 0006917 |
|  | 0008624 |
|  | 0019215 |
|  | 0004872 |
|  | 0042802 |
|  | 0005515 |
|  | 0005123 |

---

|  |  |
| --- | --- |
| KEGG ID | hsa:8717 |
|  | mmu:71609 |
|  | mmu:105242493 |
|  | rno:246756 |

---

|  |  |
| --- | --- |
| MedLine Reference | 12143389 |
|  | 10349636 |
|  | 10442092 |
|  | 10702415 |
|  | 11042159 |
|  | 11076861 |
|  | 11217851 |
|  | 11241200 |
|  | 12045187 |
|  | 12466851 |
|  | 12477932 |
|  | 12819136 |
|  | 14730360 |
|  | 15537664 |
|  | 15761471 |
|  | 15782199 |
|  | 16141072 |
|  | 16141073 |
|  | 8565075 |
|  | 8844497 |
|  | 8889548 |
|  | 9620659 |
|  | 9916988 |
|  | 10187805 |
|  | 10400625 |
|  | 10848577 |
|  | 10892748 |
|  | 10911999 |
|  | 11032752 |
|  | 11112773 |
|  | 11435712 |
|  | 11684708 |
|  | 12417987 |
|  | 12446787 |
|  | 12576460 |
|  | 12604596 |
|  | 12665801 |
|  | 12761501 |
|  | 12887920 |
|  | 12911633 |
|  | 14585990 |
|  | 14660619 |
|  | 14702039 |
|  | 16006552 |
|  | 16227624 |
|  | 16344560 |
|  | 16611992 |
|  | 7758105 |
|  | 8612133 |
|  | 8681376 |
|  | 8702708 |
|  | 8934525 |
|  | 8943045 |
|  | 9356494 |
|  | 9427646 |
|  | 9430227 |
|  | 9714541 |
|  | 9916731 |

---

|  |  |
| --- | --- |
| Organism | Homo sapiens {Organism urn:agi-taxid:9606} |
|  | Mus musculus {Organism urn:agi-taxid:10090} |
|  | Rattus norvegicus {Organism urn:agi-taxid:10116} |
|  | Homo sapiens |
|  | Mus musculus |
|  | Rattus norvegicus |

---

|  |  |
| --- | --- |
| Mouse chromosome position | 8 53.04 cM |
|  | 8 51.0 cM |

---

|  |  |
| --- | --- |
| OMIM ID | 603500 |

---

|  |  |
| --- | --- |
| Rat chromosome position | 19q11 |

---

|  |  |
| --- | --- |
| Hugo ID | 12030 |
|  | HGNC:12030 |

---

|  |  |
| --- | --- |
| Human chromosome position | 16q22.1 |
|  | 16q22 |

---

|  |  |
| --- | --- |
| Swiss-Prot Accession | Q15628 |
|  | Q15628.2 |
|  | B2RRZ7 |
|  | Q3U0V2 |
|  | Q3U0V2.1 |
|  | D3ZZC5 |
|  | B2RDS3 |
|  | B3KQZ9 |
|  | Q52NZ1 |
|  | Q8K3Z8 |
|  | TRADD\_HUMAN |
|  | TRADD\_MOUSE |
|  | B2RDS3\_HUMAN |
|  | B2RRZ7\_MOUSE |

---

|  |  |
| --- | --- |
| PIR ID | A56911 |

---

|  |  |
| --- | --- |
| GenBank ID | NC\_000016 |
|  | NM\_003789 |
|  | NP\_003780 |
|  | NM\_001323552 |
|  | NP\_001310481 |
|  | XM\_005256213 |
|  | XP\_005256270 |
|  | XM\_017023815 |
|  | XP\_016879304 |
|  | AC074143 |
|  | AJ307882 |
|  | AJ311614 |
|  | CAC38018 |
|  | AJ311615 |
|  | AJ311616 |
|  | AY575851 |
|  | AAS68637 |
|  | AY995114 |
|  | AAX89407 |
|  | CH471092 |
|  | EAW83081 |
|  | HD119556 |
|  | CBW52910 |
|  | AK090673 |
|  | BAG52211 |
|  | AK301593 |
|  | BAG63082 |
|  | AK315654 |
|  | BAG38020 |
|  | BC004491 |
|  | AAH04491 |
|  | BG717460 |
|  | BQ707739 |
|  | BT006934 |
|  | AAP35580 |
|  | CA426418 |
|  | DA353575 |
|  | L41690 |
|  | AAA98482 |
|  | Q15628 |
|  | NC\_000074 |
|  | NM\_001033161 |
|  | NP\_001028333 |
|  | AC124584 |
|  | AC124713 |
|  | CH466525 |
|  | EDL11255 |
|  | AK018592 |
|  | AK156540 |
|  | BAE33749 |
|  | AK178733 |
|  | AK191671 |
|  | BC028542 |
|  | AAH28542 |
|  | BC132607 |
|  | AAI32608 |
|  | BC138642 |
|  | AAI38643 |
|  | Q3U0V2 |
|  | NC\_005118 |
|  | XM\_006255444 |
|  | XP\_006255506 |
|  | XM\_006255442 |
|  | XP\_006255504 |
|  | NM\_001100480 |
|  | NP\_001093950 |
|  | AC\_000087 |
|  | AAHX01098146 |
|  | AC120484 |
|  | CH473972 |
|  | EDL92353 |
|  | AF517017 |
|  | AAM74196 |
|  | FQ215613 |
|  | XM\_011523420 |
|  | XP\_011521722 |
|  | NC\_018927 |
|  | AMYH02032225 |
|  | DQ893419 |
|  | ABM84345 |
|  | DQ896738 |
|  | ABM87737 |
|  | AC\_000030 |
|  | AAHY01074160 |
|  | AC\_000148 |
|  | ABBA01025431 |
|  | AAHY01074161 |
|  | AAHY01074162 |
|  | XM\_006255443 |
|  | XP\_006255505 |
|  | AABR06098507 |
|  | NT\_010498 |
|  | NW\_001838290 |
|  | NW\_926462 |
|  | NG\_009294 |
|  | AC\_000059 |
|  | NT\_078575 |
|  | NW\_001030904 |
|  | NW\_001084744 |
|  | NW\_047535 |
|  | Q8K3Z8 |
|  | XM\_341671 |
|  | XP\_341672 |
|  | XM\_001071920 |
|  | XP\_001071920 |
|  | NP\_700474 |
|  | Q52NZ1 |
|  | AA930854 |
|  | NM\_153425 |
|  | AA201054 |
|  | NW\_000349 |
|  | BB749262 |
|  | AW121848 |
|  | 21668024 |
|  | 81866722 |
|  | 123790740 |
|  | 124376502 |
|  | 74224347 |
|  | 119603487 |
|  | 124000457 |
|  | 13325367 |
|  | 14970570 |
|  | 30582707 |
|  | 45599142 |
|  | 6094511 |
|  | 62632869 |
|  | 74725760 |
|  | 808915 |

---

|  |  |
| --- | --- |
| Swiss-Prot ID | TRADD\_HUMAN |
|  | TRADD\_MOUSE |
|  | D3ZZC5\_RAT |
|  | B2RRZ7\_MOUSE |
|  | B2RDS3\_HUMAN |

---

|  |  |
| --- | --- |
| Cell Localization | Nucleus |
|  | Cytoplasm |
|  | Cytoskeleton |
|  | Cytoplasm, cytoskeleton |

---

|  |  |
| --- | --- |
| Ensembl ID | ENSG00000102871 |
|  | ENSP00000341268.4 |
|  | ENST00000345057.9 |
|  | ENSP00000462591.1 |
|  | ENST00000486556.1 |
|  | ENSMUSG00000031887 |
|  | ENSMUSP00000034359.3 |
|  | ENSMUST00000034359.9 |
|  | ENSRNOG00000015179 |
|  | ENSRNOP00000020405.4 |
|  | ENSRNOT00000020405.6 |
|  | ENSP00000341268 |
|  | ENST00000345057 |
|  | ENSP00000462591 |
|  | ENST00000486556 |
|  | ENSMUSP00000034359 |
|  | ENSMUST00000034359 |
|  | ENSRNOP00000020405 |
|  | ENSRNOT00000020405 |

---

|  |  |
| --- | --- |
| MGI ID | MGI:109200 |
|  | 109200 |
|  | 2142447 |

---

|  |  |
| --- | --- |
| RGD ID | 621313 |

---

|  |  |
| --- | --- |
| Unigene ID | Hs.460996 |
|  | Mm.264255 |
|  | Rn.18545 |

---

|  |  |
| --- | --- |
| Homologene ID | 2807 |

---

|  |  |
| --- | --- |
| IPI ID | IPI00018744 |
|  | IPI00153113 |
|  | IPI00202783 |
|  | IPI00374856 |
|  | IPI00880652 |
|  | IPI00880553 |
|  | IPI00778766 |

---

|  |  |
| --- | --- |
| Homo sapiens Chromosome position | 16q22 |

---

|  |  |
| --- | --- |
| Ensembl Transcript ID | ENST00000345057 |

---

|  |  |
| --- | --- |
| FunctionalClass | death receptor binding |
|  | protein binding |
|  | identical protein binding |
|  | signal transducer |

---

|  |  |
| --- | --- |
| Mus musculus Chromosome position | 8 51.0 cM |
|  | 8 |

---

|  |  |
| --- | --- |
| KEGG pathway | Adipocytokine signaling pathway |
|  | Apoptosis |

---

|  |  |
| --- | --- |
| Rattus norvegicus Chromosome position | 19q11 |

---

|  |  |
| --- | --- |
| Ensembl Gene ID | ENSG00000102871 |

---
